# Identification of SLAMF1 as an immune-related key gene associated with rheumatoid arthritis and verified in mice collagen-induced arthritis model

**DOI:** 10.3389/fimmu.2022.961129

**Published:** 2022-08-30

**Authors:** Anqi Li, Zhanfeng Zhang, Xiaochen Ru, Yanfeng Yi, Xiaoyu Li, Jing Qian, Jue Wang, Xiaobing Yang, Yunliang Yao

**Affiliations:** ^1^ School of Medicine & Nursing, Huzhou University, Huzhou, China; ^2^ The First Affiliated Hospital, Huzhou University, Huzhou, China; ^3^ School of Science and Engineer of Huzhou College, Huzhou, China; ^4^ Department of Rheumatology, Huzhou Third Municipal Hospital, Huzhou, China

**Keywords:** SLAMF1, rheumatoid arthritis, collagen-induced arthritis, bioinformatics, animal model, key gene

## Abstract

**Background:**

Rheumatoid arthritis (RA) is the most common inflammatory arthropathy. Immune dysregulation was implicated in the pathogenesis of RA. Thus, the aim of the research was to determine the immune related biomarkers in RA.

**Methods:**

We downloaded the gene expression data of RA in GSE89408 and GSE45291 from Gene Expression Omnibus public database (GEO). Differentially expressed genes (DEGs) were identified between RA and control groups. Infiltrating immune cells related genes were obtained by ssGSEA and weighted gene co-expression network analysis (WGCNA). We performed functional enrichment analysis of differentially expressed immunity-related genes (DEIRGs) by “clusterProfiler” R package, key genes screening by protein-protein interaction (PPI) network of DEIRGs. And mice collagen-induced arthritis (CIA) model was employed to verify these key genes.

**Results:**

A total of 1,885 up-regulated and 1,899 down-regulated DEGs were identified in RA samples. The ssGSEA analysis showed that the infiltration of 25 cells was significantly different. 603 immune related genes were obtained by WGCNA, and 270 DEIRGs were obtained by taking the intersection of DEGs and immune related genes. Enrichment analyses indicated that DEIRGs were associated with immunity related biological processes. 4 candidate biomarkers (*CCR7, KLRK1, TIGIT* and *SLAMF1*) were identified from the PPI network of DEIRGs and literature research.

In mice CIA model, the immunohistochemical stain showed *SLAMF1* has a significantly high expression in diseased joints. And flow cytometry analysis shows the expression of *SLAMF1* on CIA mice-derived CTL cells, Th, NK cells, NKT cells, classical dendritic cell (cDCs) and monocytes/macrophages was also significantly higher than corresponding immune cells from HC mice.

**Conclusion:**

Our study identified *SMLAF1* as a key biomarker in the development and progression of RA, which might provide new insight for exploring the pathogenesis of RA.

## Introduction

Rheumatoid arthritis (RA) is a systemic autoimmune disease characterized by inflammatory changes in the synovial tissue of joints. The estimated global RA prevalence was 0.46%, accounting for 36 million people worldwide (1, 2). RA primarily during the productive years of adulthood, between the ages of 20 and 40 years, and it is more prevalent among women in developed countries (3). Eventually, RA causes irreversible damage to the joint, and it is an enormous burden on patients and society. The complications associated with RA, like respiratory problems and heart diseases and, can predispose to increased mortality (4).

Serological autoantibodies are crucial indicators in the diagnosis and prognosis of RA, the anti-immunoglobulin G (IgG) antibodies, including rheumatoid factor (RF) and anti-cyclic citrullinated peptide antibody (anti-CCP), have been used as preferred RA diagnostic criteria for decades (5). Whereas, when serum RF is negative in early onset of RA and can also be found in other autoimmune and inflammatory diseases, And anti-CCP is a highly specific but moderate sensitivity indicator for RA (6–8). Therefore, a multi-dimensional diagnosis system including more novel and effective auxiliary biomarkers is urgently needed for more accurate prediction and treatment of RA.

Studies have reported that some biomarkers, including cytokines, microRNA, and autoantibodies, may effectively diagnose early RA (9–12). Also, some works proved microbiota and metabolites maybe potential ways for RA prognosis and diagnosis (13–15). Moreover, with the clinical applications of flow cytometry, some membrane proteins have been considered to have potential as a diagnostic marker such as CD146, CD64 and CD154 (16–18).However, most biomarkers are not validated in prospective cohorts, or their clinical relevance is unclear.

RA has been recognized as a chronic immune-mediated disease resulting from combined genetic and environmental factors. Further, the malfunction of multiple immune cell types and signaling networks elicit a maladaptive tissue repair process, leading to tissue damage predominantly in the joints. RA is closely associated with various immune cells either residing in the synovium or circulating in peripheral blood, and each cell type contributes differently to the disease pathogenesis (19–21). RA runs through a relatively prolonged immune disorder stage, and the molecular characterization of this stage has the potential to identify upstream molecular targets. So, the immune system could be re-engineered to halt the disease process prior to irreversible tissue damage.

Recently, transcriptomic and analyses have been widely used to identify novel biomarkers in the diagnosis and treatment of RA. With high-throughput sequencing and bioinformatics analyses, it is possible to explore potential vital genes and signal pathways closely related to disease development. In this study, we initially selected some new hub genes in RA by downloading and re-analyzing datasets GSE45291 and GSE89408. Then we employed collagen-induced arthritis (CIA) mice as a RA model and verified signaling lymphocytic activation molecule family 1 (*SLAMF1, CD150*) as an immune-related key gene in RA using Flow Cytometry, immunohistochemistry, and real-time PCR. This work may shed light on the mechanisms of RA development at the transcriptome level and provide a new target for diagnosing, preventing, and treating RA.

## Materials and methods

### Data source

Two gene expression profiles of RA, GSE89408 and GSE45291, were identified after searching GEO. The GSE89408 data file including 152 RA samples and 28 normal samples was used as a training set. And GSE45291 including 493 RA samples and 20 normal samples was used as an external validation set.

### Identification of DEGs

R software was used to process data using the “limma-voom” package (PMID: 34616422). The log transformation was applied to the original data. statistical methods p-value and false discovery rate (FDR) were initially used and results with adj.p value < 0.05 and |log2FC| > 1.5 were regarded as significant. The analysis results were presented by volcano plot and heatmap.

### Functional and pathway enrichment analysis

The “clusterProfiler” R package (PMID: 22455463) was utilized to understand the functional and pathway enrichment information on the gene of interest. Gene Ontology (GO) analysis is a commonly used approach for annotating genes and gene products with functions including biological pathways (BP), cellular components (CC) and molecular function (MF). Kyoto Encyclopedia of Genes and Genomes (KEGG) is a database resource for understanding gene functions and utilities of the biological system. GO terms or KEGG pathways with adj.p value < 0.05 were considered statistically significant.

### Analysis of immune cell characteristics

The composition of 28 immune cell (activated B cell, activated CD4 T cell, activated CD8 T cell, activated dendritic cell, CD56^bright^ natural killer cell, CD56^dim^ natural killer cell, central memory CD4 T cell, central memory CD8 T cell, effector memory CD4 T cell, effector memory CD8 T cell, eosinophil, gamma delta T cell, immature B cell, immature dendritic cell, macrophage, mast cell, MDSC, memory B cell, monocyte, natural killer cell, natural killer T cell, neutrophil, plasmacytoid dendritic cell, regulatory T cell, T follicular helper cell, Type 1 T helper cell, Type 17 T helper cell and Type 2 T helper cell) types in GSE89408 cohort was determined by the ssGSEA (Single-sample gene set enrichment analysis) method. ssGSEA was performed by “GSVA” R package (PMID: 23323831). The difference in the immune cell infiltration between the RA and normal groups was carried out using the Wilcoxon rank-sum test (p value < 0.05).

### Weighted gene co-expression network construction and analysis

The WGCNA was used to determine the relationship between co-expression gene modules and differentially infiltrating immune cells in the R package “WGCNA” (PMID: 19114008). The WGCNA network and the co-expressed gene modules were established and detected using the soft threshold power of β = 12. The genes in the core module were selected for further analyses.

### Construction of PPI network and identification of hub genes

The protein-protein interaction (PPI) of DEIRGs was obtained using the Search Tool for the Retrieval of Interacting Genes database (STRING, PMID: 12519996) and visualized by Cytoscape software (PMID: 14597658). The Core module in the PPI network was extracted by Molecular Complex Detection (MCODE, PMID: 32684073) plug-in. The parameters were set as: degree cutoff = 2, K-Core = 2, and Node Score Cutoff = 0.2. The genes in the core module were regarded as the potential hub genes.

### Validation of SLAMF1 by ROC curve

Receiver operating characteristic (ROC) was used to evaluate the ability of SLAMF1 to discriminate between RA and control samples. And the expression and discriminating ability of SLAMF1 were also tested in the external validation set. “PRROC” R package was used to calculate the precision and recall, and draw the PR curve of SLAMF1.

### Induction of collagen-induced arthritis (CIA) in mice

For CIA induction, Female DBA/1 mice were injected subcutaneously at the base of the tail with 100 µg of Chick type II collagen (Condrex, Seattle, WA, USA) emulsified in Freund’s complete adjuvant (CFA) (Chondrex, Seattle, WA, USA), followed 14 days later by a booster injection of the same Chick type II collagen (100 µg) emulsified in Freund’s incomplete adjuvant (Chondrex, Seattle, WA, USA), and mice injected with CFA/IFA only as a negative control. Which *via* the same route and following the protocol described by David et al (22). To assess the severity of arthritis, clinical symptoms were evaluated by means of a five-point scale: grade 0 = no swelling; grade 1 = paw with detectable swelling in a single digit; grade 2 = paw with swelling in more than one digit; grade 3 = paw with swelling of all digits and instep; and grade 4 = severe swelling of the paw and ankle.

### Flow cytometry

Single-cell suspensions were prepared from the peripheral blood of mice using standard procedures. Following red blood cell lysis, Fc receptors were blocked with anti-CD16/32 Ab (2.4G2), and cells were incubated with antibody in 0.5% FBS in PBS for 30 min, and then washed in FACS buffer before analysis. Data were collected on a FACSCanto™ II (BD Biosciences San Jose, CA, USA) instrument and analyzed using FlowJo software (Tree Star, Ashland, OR, USA). The Antibodies used for staining are as follows. FITC-conjugated anti-mouse CD4 (GK1.5), NKp46 (29A1.4), Siglec-F (S17007L), F4/80 (BM8), PE-conjugated anti-mouse T cell receptor γ/δ (GL3), CXCR5 (L138D7), α-GalCer : CD1d complex (L363), Gr-1 (RB6-8C5), 317 (927), PE/Cy7-conjugated anti-mouse CD8a (53-6.7), CD25 (3C7), B220 (RA3-6B2), CD11b (M1/70), CD11c (N418), APC-conjugated anti-mouse Slamf1 (W19132B) were purchased from BioLegend (San Diego, CA, USA).

### RNA extraction and real‐time PCR analysis

RNA of peripheral blood mononuclear cells was extracted by EZ-10 Total RNA Mini-Preps Kit (Sangon Biotech, Shanghai, China) and subjected to reverse transcription using HiFiScript cDNA Synthesis Kit (CoWin Biosciences, Beijing, China). The messenger RNA levels were assessed by quantitative real‐time PCR using UltraSYBR Mixture (CoWin Biosciences, Beijing, China). β-actin was used as the internal control. The primers used in the study were as follows: *β-actin*, 5′‐AGA GGG AAA TCG TGC GTG AC‐3′ (sense) and 5′‐CAA TAG TGA TGA CCT GGC CGT‐3′ (antisense); *CCR7*, 5′‐TGT ACG AGT CGG TGT GCT TC ‐3′ (sense) and 5′‐GGT AGG TAT CCG TCA TGG TCT TG‐3′ (antisense); *KLRK1*, 5′‐ACT CAG AGA TGA GCA AAT GCC‐3′ (sense) and 5′‐CAG GTT GAC TGG TAG TTA GTG C‐3′ (antisense); *TIGIT*, 5′‐ CCA CAG CAG GCA CGA TAG ATA ‐3′ (sense) and 5′‐CCA CCA CGA TGA CTG CTG T‐3′ (antisense); *Slamf1*, 5′‐CAG AAA TCA GGG CCT CAA GAG‐3′ (sense) and 5′‐ CAT GCC ACC CCA GGT CAA C ‐3′(antisense).

### Statistical analyses

Data are presented as mean ± SD. The Wilcoxon rank-sum test was used for all statistical analyses between two conditions. A P value < 0.05 was considered statistically significant (*P < 0.05; **P < 0.01; ***P < 0.001).

## Results

### Identification DEGs and the immune infiltration in RA

**Figure 1:**
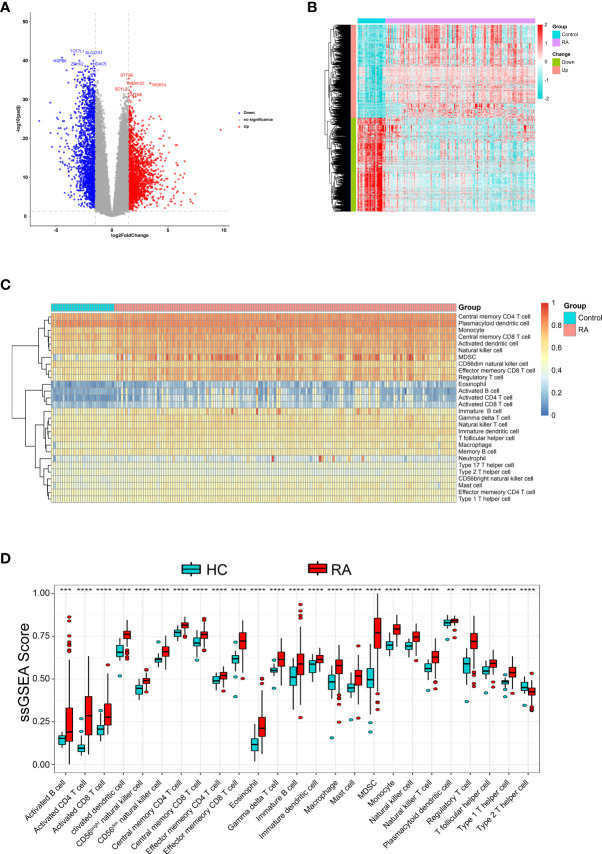
Identification DEGs and the immune infiltration in RA. **(A)** The volcano plot and **(B)** heatmap to show significant DEGs between RA and HC samples **(C)** Heatmap of the immune infiltration profiles of RA and HC samples analyzed by ssGSEA score-based method. **(D)** Comparison of immune cell infiltration between RA and HC samples. **p < 0.01, ***p < 0.001 and ****p < 0.0001.

First, GSE89408 was analyzed using the “limma-voom” package to identify DEGs. After standardization analysis, 3,784 DEGs were identified, including 1,885 up-regulated DEGs and 1,899 down-regulated DEGs (**Figures 1A, B** **)** in RA samples compared to normal samples.

Further, We analyzed whether RA and healthy control (HC) had different immune cell infiltration profiles using ssGSEA. **Figure 1C** shows the distribution of 28 infiltrating immune cells in the RA and normal samples. The Wilcoxon rank-sum test result showed that there were significant differences in the content of the 25 immune cells between RA and normal groups (**Figure 1D**), specifically, activated dendritic cell, monocyte, central memory CD4 T cell, MDSC, activated CD4 T cell, effector memory CD8 T cell, regulatory T cell, type 1 T helper cell, CD56dim natural killer cell, eosinophil, CD56bright natural killer cell, natural killer cell, natural killer T cell, activated CD8 T cell, gamma delta T cell, macrophage, T follicular helper cell, central memory CD8 T cell, mast cell, immature dendritic cell, type 2 T helper cell, immature B cell, effector memory CD4 T cell, activated B cell and plasmacytoid dendritic cell.

### WGCNA and identification of the key module associated with immune infiltration in RA

**Figure 2:**
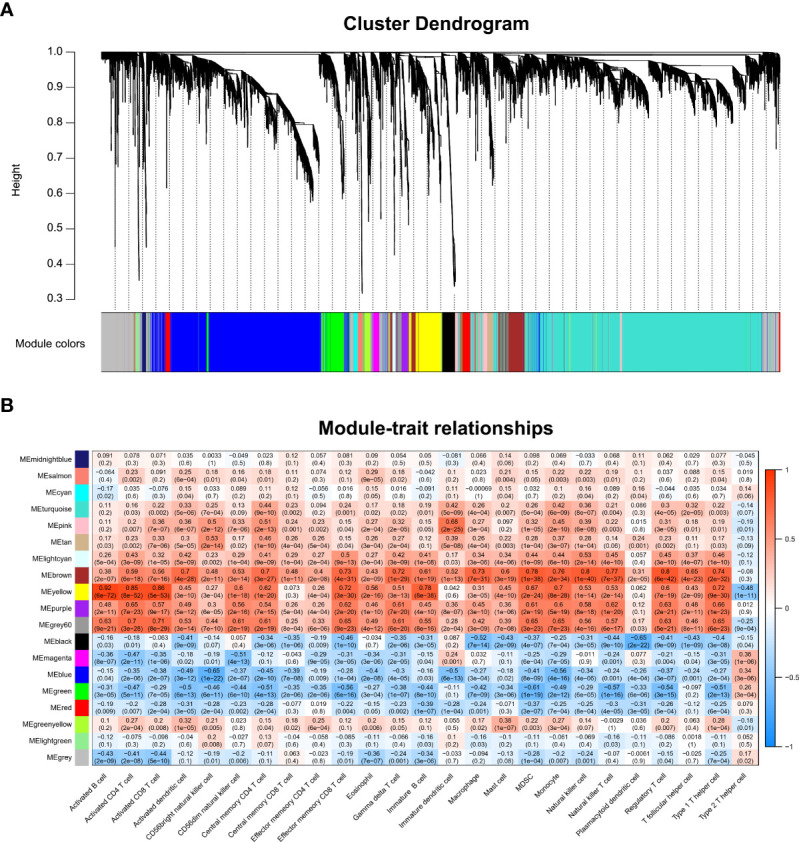
WGCNA and identification of the key module associated with immune infiltration in RA. **(A)** Clustering dendrograms for the 3,784 DEGs with dissimilarity based on the topological overlap together with the assigned module colors; 19 co-expression modules were constructed with various colors. **(B)** Heatmap of the correlation between module eigengene and differentially infiltrated immune cells. Each row represents a color-coded module eigengene, each column represents a type of infiltrated immune cells. The number in each cell means the correlation coefficient and p-value.

We performed WGCNA to screen genes associated with differentially infiltrating immune cells. **Supplementary Figure 1A** shows that the GSM2371137 sample was an outlier removed from the subsequent WGCNA. The clustering of the remaining samples is shown in **Supplementary Figure 1B**. β value 12 was chosen as the appropriate soft-thresholding value (**Supplementary Figure 1C**). The WGCNA detected 19 co-expression modules, and the cluster dendrogram was established (**Figure 2A**). Then, their correlations with differentially infiltrating immune cells were analyzed. The yellow module was mainly associated with immune cells such as lymphocytes, including activated B cells, activated CD4 T cells, and activated CD8 T cells (|Cor| > 0.8, p-value < 0.05, **Figure 2B**). We defined the yellow module as an immune-related module, and a total of 603 genes in this module were extracted for subsequent studies.

### Enrichment and identification analysis of hub genes

**Figure 3:**
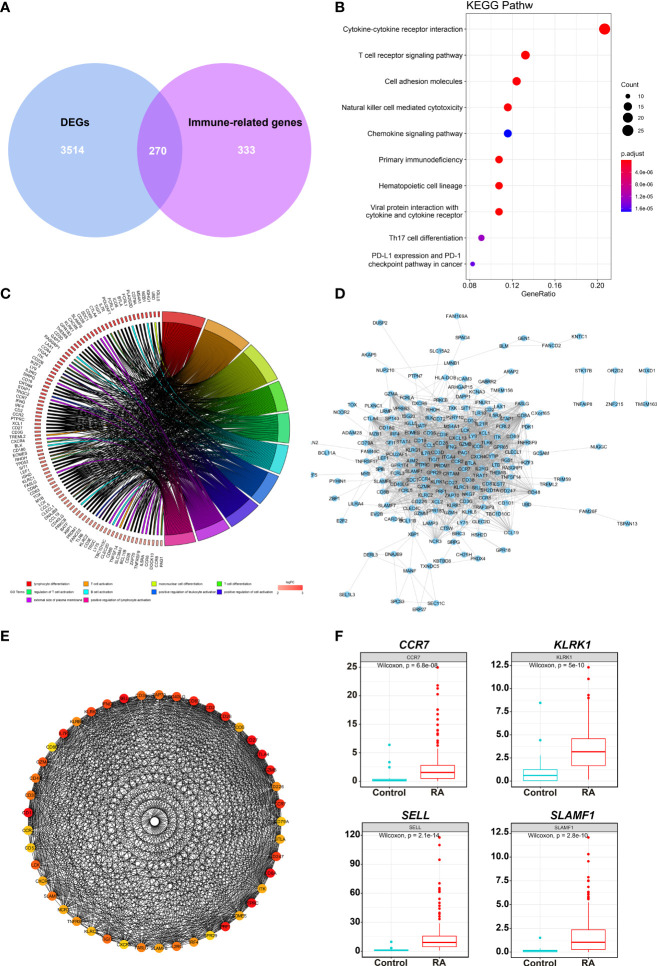
DEIRGs screening and enrichment analysis. **(A)** Venn diagram of 270 overlapped candidate genes shared by DEGs and Immune-related genes (MEyellow module). KEGG **(B)** and GO **(C)** enrichment analysis showed the most significantly enriched pathways. **(D)** Protein-protein interaction (PPI) analysis by STRING database for 270 DEIRGs. **(E)** PPI diagram of all the genes in cluster 1. **(F)** Expression levels of 4 potential biomarkers between RA and HC samples.

A total of 270 DEIRGs were obtained by intersecting 603 module genes with 3,784 DEGs (**Figure 3A**). The most enriched KEGG pathways were “cytokine-cytokine receptor interaction,” “T cell receptor signaling pathway,” “cell adhesion molecules”, “natural killer cell-mediated cytotoxicity”, “chemokine signaling pathway”, “primary immunodeficiency” (**Figure 3B**). According to GO enrichment analysis, the most significantly enriched terms were “lymphocyte differentiation,” “T cell activation,” “immunological synapse”, “T cell receptor complex”, “immune receptor activity” “cytokine receptor activity” (**Figure 3C**).

The PPI was analyzed with 270 DEIRGs using the STRING databas**e** (**Figure 3D**). The interactive relationships between the key genes in the whole network were determined using the Cytoscape plugin MCODE. There were 6 clusters: 45 nodes and 744 edges in cluster 1 (**Figure 3E**), 17 nodes and 36 edges in cluster 2, 4 nodes and 6 edges in cluster 3, 4 nodes and 5 edges in cluster 4, 3 nodes and 3 edges in cluster 5, and 3 nodes and 3 edges in cluster 6 (**Supplementary Figure 2**). Then, 27 hub TOP genes degree score≥30 in cluster 1 was used to identify hub genes.

We conducted literature research to find novel immune-related RA diagnostic biomarkers. We eliminated the genes encoding classical immune cell markers and genes with a clear function in or associated with RA. We identified 4 genes as candidate RA biomarkers for further validation, including *CCR7, KLRK1, TIGIT*, and *SLAMF1* (**Supplementary Table 1**). **Figure 3F** shows the expression levels of the above genes in the RA samples, and they were significantly higher than those in the control group.

### Validation of candidate biomarkers in mice model

**Figure 4:**
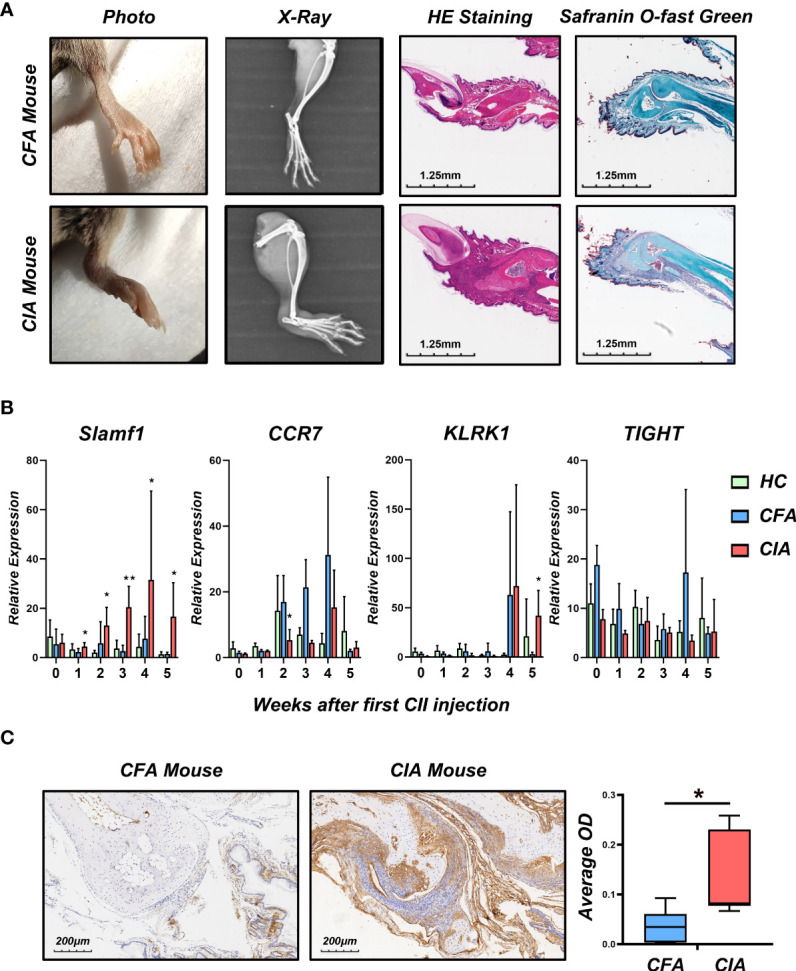
Construction collagen-induced arthritis and validation of candidate gene in the model. **(A)** Photographs, X-ray, H&E and Safranin O-fast green staining images of the hind limbs of CIA and HC 5 weeks after the first CII injection. **(B)** Realtime-PCR analysis for gene expression of *CCR7, KLRK1, TIGIT and SLAMF1* in the PBMC of CIA, CFA control and healthy control (HC) mice every week. **(C)** Immunohistochemical detection of *SLAMF1* expression in joint synovial samples from CIA and CFA mice. Data shown are the mean ± SEM with 6 mice per group and are from one experiment, representative of two performed. *p < 0.05 and **p < 0.01 (Wilcoxon rank-sum test).

To verify the role of candidate genes in RA, we employed classical collagen-induced arthritis (CIA) model in DBA/1 mice using subcutaneous injection of chick type II collagen. We observed a perfect joint pathological damage in this animal model (**Figure 4A**). To dynamically detect the expression of candidate genes in the pathological process, we isolated PBMCs from Healthy Control mice (HC), Freund’s Complete Adjuvant injected mice (CFA Control) and CIA mice every week. We detected the mRNA expression level of *CCR7, KLRK1, TIGIT*, and *SLAMF1* and found that *SLAMF1* has the most consistent trend of significance with the pathological progression of CIA (**Figure 4B**). Further, the immunohistochemical stain of joint tissue showed *SLAMF1* has a significantly high expression in diseased joints (**Figure 4C**), suggesting that *SLAMF1* might be involved in the immune response caused by multiple immune cell infiltration.

**Figure 5:**
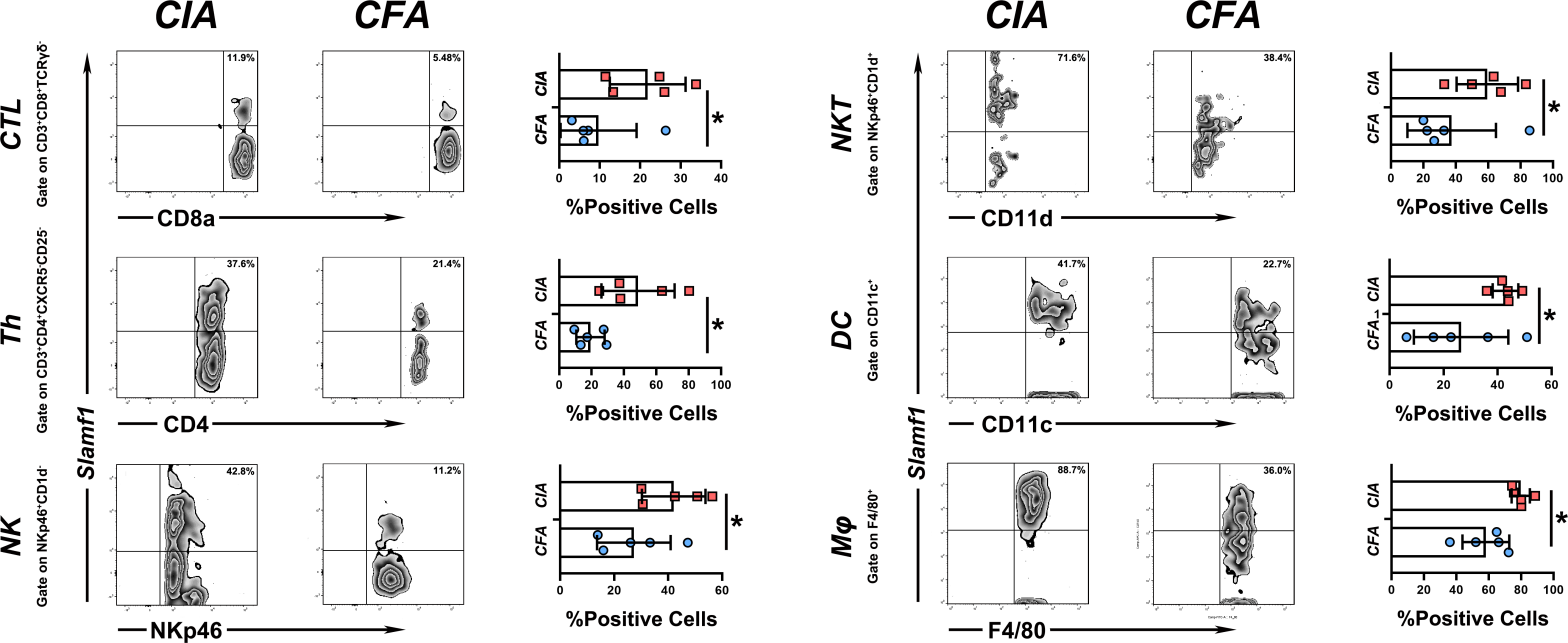
Expression level of slamf1 on different immune cell subsets PBMC from CIA and CFA mice were stained with respective surface markers and grouped into different immune cell subsets, the expression of *SLAMF1* were detected by flow cytometry and analysis by mean fluorescence intensity (MFI). Data shown are the mean ± SEM with 6 mice per group and are from one experiment, representative of two performed. *p <0.05, **p < 0.01 and ***p < 0.001 (Wilcoxon rank-sum test).

Since *SLAMF1* is a typical membrane protein and is also known as CD150, we used flow cytometry to investigate the role of *SLAMF1* in the RA-related immune responses and detected the expression of *SLAMF1* on the surface of immune cells of CIA and HC mice using ssGSEA as mentioned above. To simplify the detection, we grouped 23 immune cells into 11 categories according to surface markers and gate strategy (**Supplementary Table 2**). We excluded plasmacytoid dendritic cell (pDCs) and mast cells from grouping as they cannot be detected in PBMCs. The results showed significantly elevated expressions of *SLAMF1* in the Cytotoxic T Cell (CTL), T helper cells (Th), Natural killer cells (NK), Natural killer T cells (NKT), classical dendritic cell (cDCs), Monocytes/Macrophages (Mφ) (**Figure 5**), But not in Tfh cells, Treg cells, B cells, Eosinophils, and MDSC (Data not shown).

### Evaluation and verification of the diagnostic effect of *SLAMF1* on RA

**Figure 6:**
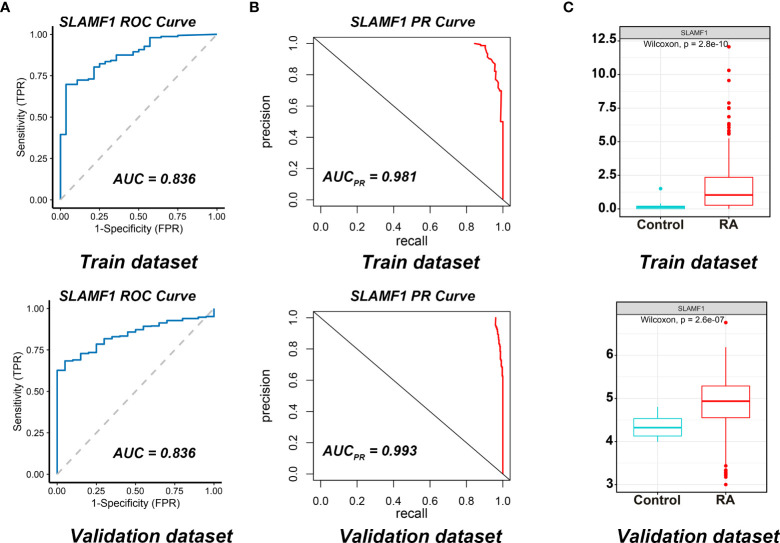
Diagnostic effect of *SLAMF1* on RA. **(A)** ROC curve and **(B)** Precision-recall evaluation of the diagnostic effectiveness of SLAMF1 biomarkers in Train Dataset (GSE89408) and Validation Dataset (GSE45291). **(C)** The expressions of *SLAMF1* in Train Dataset (GSE89408) and Validation Dataset (GSE45291).

We used the ROC curves to assess *SLAMF1* diagnostic efficiency and determine whether it has an excellent diagnostic value in RA patients. As showed in **Figures 6A, B**, the AUCROC and AUCPR of Slamf1 were 0.899 and 0.981, also which indicated that *SLAMF1* has a particular reference value in diagnosing RA patients.

We further verified the expressions and diagnostic values of *SLAMF1* using the GSE45291 as a validation dataset. We found that the expression trends (**Figure 6C**) and the AUCs (**Figures 6A, B**) were consistent with those in the training set GSE89408.

## Discussion

Rheumatoid arthritis (RA), characterized by joint inflammation leading to progressive tissue damage and joint disability, is among the most frequent chronic inflammatory diseases (23). In the last decade, from a microscopic to a macroscopic level, we have seen unprecedented significant insight into the cellular function and molecular mechanisms involved with RA. However, the pathogenesis of RA remains incompletely understood, and researching the key genes underlying RA development remains a priority. Our previous studies found that Thymocyte-expressed positive selection-associated 1 (*Tespa1*) plays a critical role in the pathogenesis of human rheumatoid arthritis and mice collagen-induced arthritis, thus defining *Tespa1* as a key gene in the pathogenesis of RA (24, 25). With the development of high-throughput sequencing and bioinformatic analytical techniques, DNA microarray has become an important method to obtain gene expression data of multiple diseases on a large scale and efficiently. Through integrated analysis of multiple sequencing databases, several studies have identified key genes and biomarkers that contribute to the occurrence and progression of RA (26–29). However, most results obtained in these studies were not physiologically validated. Therefore, further studies are required to screen and verify biomarkers involved in the pathogenesis of RA more precisely and reliably.

The present study aimed to identify and verify novel biomarkers highly expressed in RA compared to normal controls and reveal their potential mechanisms. We extracted the gene expression profiles of GSE89408, and GSE45291 downloaded from the GEO database and identified 1,885 up-regulated and 1,899 down-regulated overlap DEGs. According to the GO functional enrichment analysis, the RA-related DEGs were primarily enriched in the chemokine-mediated signaling pathway, T cell activation and regulation of T cell activation in BP; external side of the plasma membrane, collagen-containing extracellular matrix, and collagen trimer in CC; and immune receptor activity, chemokine activity and cytokine activity in MF. KEGG pathway analysis showed that the DEGs were enriched in cytokine-cytokine receptor interaction and chemokine signaling pathway viral protein interaction with cytokines. These functional enrichment analyses indicated that the DEGs were enriched in immune response and inflammation, which play an essential role in the pathogenesis of RA (20).

Increasing evidence shows that various immune cells play pathogenic roles in RA (19, 30). This study mapped the roles of various immune cells in the pathological process of RA. We analyzed the infiltration profiles of 28 infiltrating immune cells using ssGSEA and found that 23 of them have a significant increase compared to healthy controls. Next, we performed WGCNA to screen genes associated with differentially infiltrating immune cells and obtained 603 immune-related genes in one module. By intersecting DEGs and immune-related modules, we screened 270 DEIRGs and finally identified *CCR7, KLRK1, TIGIT*, and *SLAMF1* as candidate RA biomarkers using literature research.

To verify the role of these genes in RA, we employed a mice CIA model to dynamically detect the expression of candidate genes in both peripheral blood mononuclear cells and pathological joint tissue. We proved that *SLAMF1* has a perfect fitting curve with the CIA disease process. We detected the expression of *SLAMF1* on different immune cells using flow cytometry. We demonstrated a significantly elevated expression of *SLAMF1* on CTLs, Th cells, NK cells, NKT cells, cDC, and Mφ. Therefore, *SLAMF1* may be involved in the inflammatory response mediated by these infiltration immune cells in the pathogenesis of RA. Finally, the ROC curves indicated that *SLAMF1* had a particular reference value in the diagnosis of RA patients, prompting that *SLAMF1* may be an essential biomarker for RA.

Nevertheless, there are several limitations to this study. Firstly, the samples in datasets GSE89408 and GSE45291 were from joint synovial biopsies and peripheral blood mononuclear cells respectively. Different specimen sources may have some impact on the subsequent analysis results. Secondly, the relationships between the biomarkers and immune activation in rheumatoid arthritis were only verified by the expression. Moreover, although the datasets we analyzed were from clinical samples, our validation work was carried out only in mouse models, we will validate these findings in clinical patient samples in future work. Finally, additional *in vitro* and *in vivo* experiments are necessary for exploring the in-depth mechanisms of how the *SLAMF1* regulates inflammatory responses in RA.

In summary, the present study revealed that *SLAMF1* might play a key role in the development and progression of RA, which might provide new insight for exploring the pathogenesis of RA, a basis for finding auxiliary diagnostic biomarker and potential therapeutic targets for RA.

## Data availability statement

The raw data supporting the conclusions of this article will be made available by the authors, without undue reservation.

## Ethics statement

The animal study was reviewed and approved by Animal ethics committee of First Affiliated Hospital of Huzhou University.

## Author contributions

AL and ZZ: Methodology, Data Curation and Writing - Original Draft. XR, YYi and XL: Investigation. JQ and JW: Validation. XY: Resources. YYa: Conceptualization, Supervision and Writing - Review & Editing.

## Funding

This work was supported by the National Natural Science of China [31870886], the Natural Science Foundation of Zhejiang Province [No. LQ20C080001], Medical Health Science and Technology Project of Zhejiang Provincial Health Commission [No.2021KY346], Huzhou Municipal Science and Technology Bureau [2021YZ26].

## Acknowledgments

Thanks to my nine-year-old daughter Qianshu Yao for her contribution in the mouse observation.

## Conflict of interest

The authors declare that the research was conducted in the absence of any commercial or financial relationships that could be construed as a potential conflict of interest.

## Publisher’s note

All claims expressed in this article are solely those of the authors and do not necessarily represent those of their affiliated organizations, or those of the publisher, the editors and the reviewers. Any product that may be evaluated in this article, or claim that may be made by its manufacturer, is not guaranteed or endorsed by the publisher.

## References

[1] van der Woude D van der Helm-van Mil AHM . Update on the epidemiology, risk factors, and disease outcomes of rheumatoid arthritis. Best Pract Res Clin Rheumatol (2018) 32(2):174–87. doi: 10.1016/j.berh.2018.10.005 30527425

[2] Almutairi KB Nossent JC Preen DB Keen HI Inderjeeth CA . The prevalence of rheumatoid arthritis: A systematic review of population-based studies. J Rheumatol (2021) 48(5):669–76. doi: 10.3899/jrheum.200367 33060323

[3] Symmons DP . Epidemiology of rheumatoid arthritis: Determinants of onset, persistence and outcome. Best Pract Res Clin Rheumatol (2002) 16(5):707–22. doi: 10.1053/berh.2002.0257 12473269

[4] Young A Koduri G Batley M Kulinskaya E Gough A Norton S . Mortality in rheumatoid arthritis. increased in the early course of disease, in ischaemic heart disease and in pulmonary fibrosis. Rheumatol (Oxford) (2007) 46(2):350–7. doi: 10.1093/rheumatology/kel253 16908509

[5] Tenstad HB Nilsson AC Dellgren CD Lindegaard HM Rubin KH Lillevang ST . Predictive values of anti-cyclic citrullinated peptide antibodies and rheumatoid factor in relation to serological aspects of the ACR/EULAR 2010 classification criteria for rheumatoid arthritis. Scand J Rheumatol (2020) 49(1):18–20. doi: 10.1080/03009742.2019.1609079 31264518

[6] Song YW Kang EH . Autoantibodies in rheumatoid arthritis: Rheumatoid factors and anticitrullinated protein antibodies. QJM (2010) 103(3):139–46. doi: 10.1093/qjmed/hcp165 PMC2825384 19926660

[7] Kroot EJ de Jong BA van Leeuwen MA Swinkels H van den Hoogen FH van't Hof M . The prognostic value of anti-cyclic citrullinated peptide antibody in patients with recent-onset rheumatoid arthritis. Arthritis Rheumatol (2000) 43(8):1831–5. doi: 10.1002/1529-0131(200008)43:8<1831::AID-ANR19>3.0.CO;2-6 10943873

[8] Curtis JR van der Helm-van Mil AH Knevel R Huizinga TW Haney DJ Shen Y . Validation of a novel multibiomarker test to assess rheumatoid arthritis disease activity. Arthritis Care Res (Hoboken) (2012) 64(12):1794–803. doi: 10.1002/acr.21767 PMC3508159 22736476

[9] Burska A Boissinot M Ponchel F . Cytokines as biomarkers in rheumatoid arthritis. Mediators Inflamm (2014) 2014:545493. doi: 10.1155/2014/545493 24733962 PMC3964841

[10] Singh A Patro PS Aggarwal A . MicroRNA-132, miR-146a, and miR-155 as potential biomarkers of methotrexate response in patients with rheumatoid arthritis. Clin Rheumatol (2019) 38(3):877–84. doi: 10.1007/s10067-018-4380-z 30511295

[11] Conti V Corbi G Costantino M De Bellis E Manzo V Sellitto C . Biomarkers to personalize the treatment of rheumatoid arthritis: Focus on autoantibodies and pharmacogenetics. Biomolecules (2020) 10(12):1672. doi: 10.3390/biom10121672 PMC7765045 33327600

[12] Luque-Tevar M Perez-Sanchez C Patino-Trives AM Barbarroja N Arias de la Rosa I Abalos-Aguilera MC . Integrative clinical, molecular, and computational analysis identify novel biomarkers and differential profiles of anti-TNF response in rheumatoid arthritis. Front Immunol (2021) 12:631662. doi: 10.3389/fimmu.2021.631662 33833756 PMC8022208

[13] Zhang X Zhang D Jia H Feng Q Wang D Liang D . The oral and gut microbiomes are perturbed in rheumatoid arthritis and partly normalized after treatment. Nat Med (2015) 21(8):895–905. doi: 10.1038/nm.3914 26214836

[14] Liu Z Wu Y Luo Y Wei S Lu C Zhou Y . Self-balance of intestinal flora in spouses of patients with rheumatoid arthritis. Front Med (Lausanne) (2020) 7:538. doi: 10.3389/fmed.2020.00538 33681234 PMC7931358

[15] Chen Y Ma C Liu L He J Zhu C Zheng F . Analysis of gut microbiota and metabolites in patients with rheumatoid arthritis and identification of potential biomarkers. Aging (Albany NY) (2021) 13(20):23689–701. doi: 10.3389/fimmu.2022.961708 PMC8580343 34670873

[16] Matsui T Ohsumi K Ozawa N Shimada K Sumitomo S Shimane K . CD64 on neutrophils is a sensitive and specific marker for detection of infection in patients with rheumatoid arthritis. J Rheumatol (2006) 33(12):2416–24. 17080517

[17] Neidhart M Wehrli R Bruhlmann P Michel BA Gay RE Gay S . Synovial fluid CD146 (MUC18), a marker for synovial membrane angiogenesis in rheumatoid arthritis. Arthritis Rheumatol (1999) 42(4):622–30. doi: 10.1002/1529-0131(199904)42:4<622::AID-ANR4>3.0.CO;2-Y 10211875

[18] Berner B Wolf G Hummel KM Muller GA Reuss-Borst MA . Increased expression of CD40 ligand (CD154) on CD4+ T cells as a marker of disease activity in rheumatoid arthritis. Ann Rheum. Dis (2000) 59(3):190–5. doi: 10.1136/ard.59.3.190 PMC1753086 10700427

[19] Yap HY Tee SZ Wong MM Chow SK Peh SC Teow SY . Pathogenic role of immune cells in rheumatoid arthritis: Implications in clinical treatment and biomarker development. Cells (2018) 7(10):161. doi: 10.3390/cells7100161 PMC6211121 30304822

[20] Weyand CM Goronzy JJ . The immunology of rheumatoid arthritis. Nat Immunol (2021) 22(1):10–8. doi: 10.1038/s41590-020-00816-x PMC8557973 33257900

[21] Firestein GS McInnes IB . Immunopathogenesis of rheumatoid arthritis. Immunity (2017) 46(2):183–96. doi: 10.1016/j.immuni.2017.02.006 PMC5385708 28228278

[22] Brand DD Latham KA Rosloniec EF . Collagen-induced arthritis. Nat Protoc (2007) 2(5):1269–75. doi: 10.1038/nprot.2007.173 17546023

[23] Smolen JS Aletaha D McInnes IB . Rheumatoid arthritis. Lancet (2016) 388(10055):2023–38. doi: 10.1016/S0140-6736(16)30173-8 27156434

[24] Yao Y Zhang H Shao S Cui G Zhang T Sun H . Tespa1 is associated with susceptibility but not severity of rheumatoid arthritis in the zhejiang han population in China. Clin Rheumatol (2015) 34(4):665–71. doi: 10.1007/s10067-015-2900-7 25736038

[25] Yao Y Huang W Li X Li X Qian J Han H . Tespa1 deficiency dampens thymus-dependent b-cell activation and attenuates collagen-induced arthritis in mice. Front Immunol (2018) 9:965. doi: 10.3389/fimmu.2018.00965 29867947 PMC5960706

[26] Yu F Hu G Li L Yu B Liu R . Identification of key candidate genes and biological pathways in the synovial tissue of patients with rheumatoid arthritis. Exp Ther Med (2022) 23(6):368. doi: 10.3892/etm.2022.11295 35495609 PMC9019691

[27] Zhang D Li ZF Zhang RQ Yang XL Zhang DN Li Q . Identification of differentially expressed and methylated genes associated with rheumatoid arthritis based on network. Autoimmunity (2020) 53(6):303–13. doi: 10.1080/08916934.2020.1786069 32650679

[28] Zhang RR Zhou XP Jin YH Chang C Wang RS Liu J . Identification of differential key biomarkers in the synovial tissue between rheumatoid arthritis and osteoarthritis using bioinformatics analysis. Clin Rheumatol (2021) 40(12):5103–10. doi: 10.1007/s10067-021-05825-1 34224029

[29] Zhu N Hou J Wu Y Li G Liu J Ma G . Identification of key genes in rheumatoid arthritis and osteoarthritis based on bioinformatics analysis. Med (Baltimore) (2018) 97(22):e10997. doi: 10.1097/MD.0000000000010997 PMC6392928 29851858

[30] Kumar H Bot A . In this issue: Role of immune cells and molecules in rheumatoid arthritis pathogenesis and cancer immunotherapy. Int Rev Immunol (2018) 37(3):127–8. doi: 10.1080/08830185.2018.1469353 29733768

