# Supplementary figures and images for: Identification of SLAMF1 as an immune-related key gene associated with rheumatoid arthritis and verified in mice collagen-induced arthritis model
Source: Front Immunol. 2022 Aug 30;13:961129. doi: 10.3389/fimmu.2022.961129 (PMC9468826; doi:10.3389/fimmu.2022.961129)

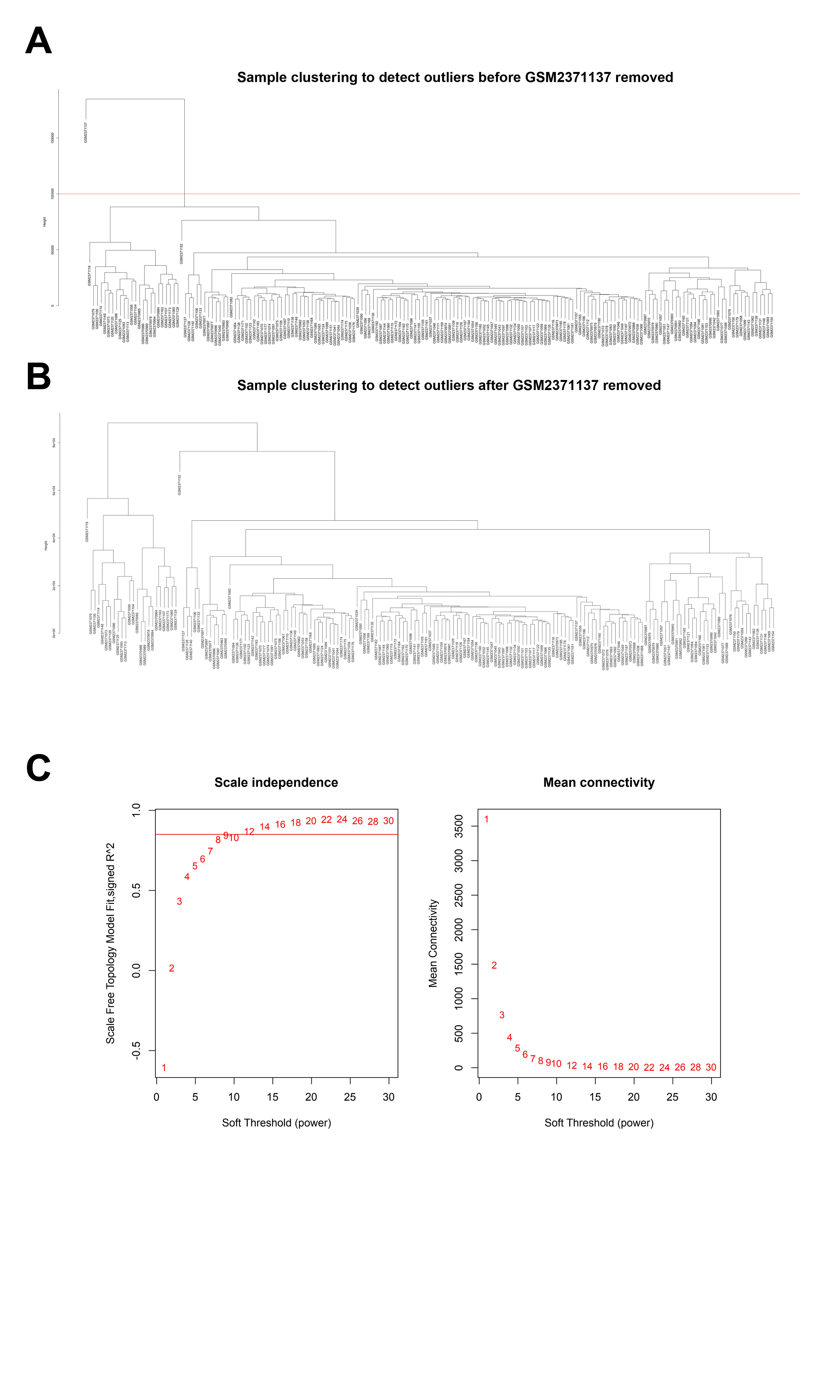

Supplement: Supplementary file 1 [file Image_1.tif]

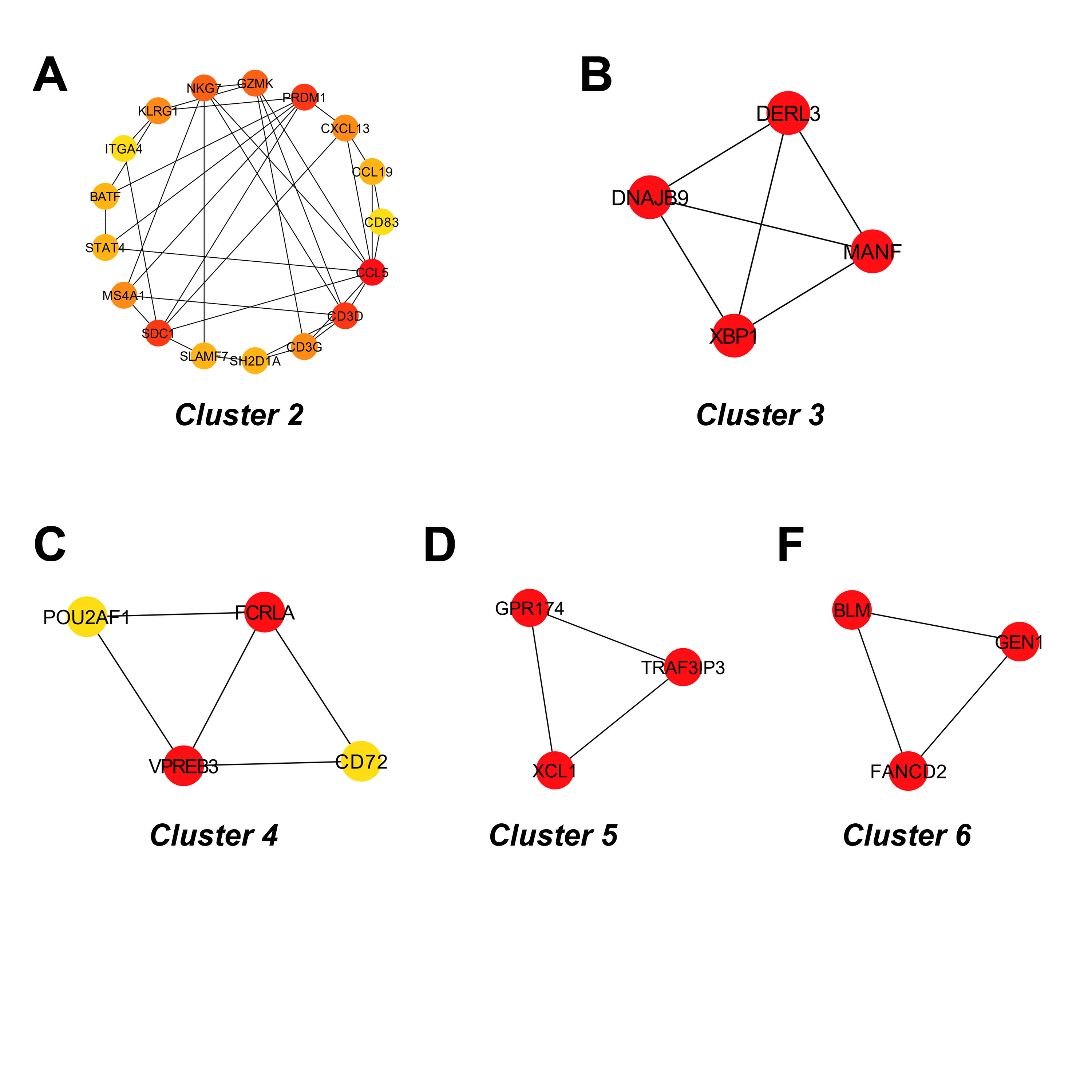

Supplement: Supplementary file 2 [file Image_2.tif]
