# Supplementary material for: Identification of SLAMF1 as an immune-related key gene associated with rheumatoid arthritis and verified in mice collagen-induced arthritis model
Source: Front Immunol. 2022 Aug 30;13:961129. doi: 10.3389/fimmu.2022.961129 (PMC9468826; doi:10.3389/fimmu.2022.961129)
Supplement: Supplementary file 3 [file Table_1.docx]

| Table 1. Screen for potential biomarkers by literature research | | | | |
| --- | --- | --- | --- | --- |
| **Ranking** | Degree score | **Gene Name** | **Reported function** | **Potential new RA biomarkers** |
| 1 | 44 | CD27 | A memory B-cell marker. ^[1,2]^ |  |
| 2 | 44 | CTLA4 | An immune checkpoint and surface marker for Treg and activated T cell^[3]^ |  |
| 3 | 44 | CD8A | A classical cytotoxic T lymphocytes (CTL) marker^[4]^ |  |
| 4 | 44 | CD19 | A B-cell marker and promising target for rheumatoid arthritis^[5,6]^ |  |
| 5 | 44 | PTPRC | A ubiquitous cell surface marker of all hematopoietic cells^[7]^ (CD45) |  |
| 6 | 44 | SELL | A late activation marker of T cell^[8]^ (CD62L)  Expression on the surface of peripheral blood leucocytes from RA patients^[9]^ |  |
| 7 | 43 | PRF1 | A pore forming cytolytic protein as a marker for CTL and NK cells^[10]^ |  |
| 8 | 43 | GZMB | A serine protease as a marker for CTL and NK cells^[11]^ |  |
| 9 | 42 | IL7R | Defined as a RA- related hub gene in numerous studies ^[12-14]^ |  |
| 10 | 42 | CD2 | A classical T cell and NK cell marker^[15]^ |  |
| 11 | 42 | CD28 | A surface marker for Naïve/ memory T cells^[16,17]^ |  |
| 12 | 42 | CD69 | A classical early marker of lymphocyte activation^[18]^ |  |
| 13 | 40 | CCR7 | A key role in migration of naïve and memory T and promising target for RA therapy in mice^[19,20]^ | Yes |
| 14 | 39 | CD247 | A classical T cell marker (CD3ζ)^[21]^ |  |
| 15 | 37 | CD40LG | Be Proved to be associated with rheumatoid arthritis clinical features^[22]^ |  |
| 16 | 37 | IFNG | IFNγ is the secretory biomarker for Th1 cells^[23,24]^ |  |
| 17 | 37 | KLRK1 | An activating receptor present on various lymphocytes result in both immune activation and immune silencing^[25]^. | Yes |
| 18 | 36 | GZMA | A serine protease as a marker for CTL and NK cells^[11]^ |  |
| 19 | 35 | LCK | Be Proved a potential role in in rheumatoid arthritis^[26]^ |  |
| 20 | 33 | TIGIT | An immune receptor presents on some T cells and natural killer cells^[27]^  TIGIT overexpression ameliorates rheumatoid arthritis in mouse models^[28]^ | Yes |
| 21 | 33 | CD38 | An activation and differentiation marker for T and B cells^[29]^ |  |
| 22 | 33 | ZAP70 | A critical component of T cell activation and development^[30]^ |  |
| 23 | 32 | SLAMF1 | A founder of signaling lymphocyte activation molecule (SLAM) family of cell-surface receptors. It is widely expressed on cells within hematopoietic system but no reports associated with RA yet^[31]^ | Yes |
| 24 | 32 | CD48 | B-lymphocyte activation marker^[32]^ |  |
| 25 | 32 | CD3E | A classical T cell marker (CD3ε)^[21]^ |  |
| 26 | 32 | IL2RG | Defined as a RA- related hub gene in numerous studies^[12,33-36]^ |  |
| 27 | 30 | FASLG | A classical apoptotic marker for T cell and reported as a susceptibility gene for RA ^[37-42]^ |  |

[1] Buchan S L, Rogel A, Al-Shamkhani A. The immunobiology of CD27 and OX40 and their potential as targets for cancer immunotherapy[J]. Blood, 2018, 131(1): 39-48.

[2] Agematsu K, Hokibara S, Nagumo H, et al. CD27: a memory B-cell marker[J]. Immunol Today, 2000, 21(5): 204-6.

[3] Ueda H, Howson J M, Esposito L, et al. Association of the T-cell regulatory gene CTLA4 with susceptibility to autoimmune disease[J]. Nature, 2003, 423(6939): 506-11.

[4] Luescher I F, Vivier E, Layer A, et al. CD8 modulation of T-cell antigen receptor-ligand interactions on living cytotoxic T lymphocytes[J]. Nature, 1995, 373(6512): 353-6.

[5] Scheuermann R H, Racila E. CD19 antigen in leukemia and lymphoma diagnosis and immunotherapy[J]. Leuk Lymphoma, 1995, 18(5-6): 385-97.

[6] Tedder T F. CD19: a promising B cell target for rheumatoid arthritis[J]. Nat Rev Rheumatol, 2009, 5(10): 572-7.

[7] Li J, Xin J, Zhang L, et al. Human hepatic progenitor cells express hematopoietic cell markers CD45 and CD109[J]. Int J Med Sci, 2014, 11(1): 65-79.

[8] Sava F, Toldi G, Treszl A, et al. Expression of lymphocyte activation markers of preterm neonates is associated with perinatal complications[J]. BMC Immunol, 2016, 17(1): 19.

[9] Bond A, Hay F C. L-selectin expression on the surface of peripheral blood leucocytes from rheumatoid arthritis patients is linked to disease activity[J]. Scand J Immunol, 1997, 46(3): 312-6.

[10] Osinska I, Popko K, Demkow U. Perforin: an important player in immune response[J]. Cent Eur J Immunol, 2014, 39(1): 109-15.

[11] Trapani J A. Target cell apoptosis induced by cytotoxic T cells and natural killer cells involves synergy between the pore-forming protein, perforin, and the serine protease, granzyme B[J]. Aust N Z J Med, 1995, 25(6): 793-9.

[12] Zhang R, Yang X, Wang J, et al. Identification of potential biomarkers for differential diagnosis between rheumatoid arthritis and osteoarthritis via integrative genomewide gene expression profiling analysis[J]. Mol Med Rep, 2019, 19(1): 30-40.

[13] Geng X D, Wang W W, Feng Z, et al. Identification of key genes and pathways in diabetic nephropathy by bioinformatics analysis[J]. J Diabetes Investig, 2019, 10(4): 972-984.

[14] Lu C, Niu X, Xiao C, et al. Network-based gene expression biomarkers for cold and heat patterns of rheumatoid arthritis in traditional chinese medicine[J]. Evid Based Complement Alternat Med, 2012, 2012: 203043.

[15] Binder C, Cvetkovski F, Sellberg F, et al. CD2 Immunobiology[J]. Front Immunol, 2020, 11: 1090.

[16] Larbi A, Fulop T. From "truly naive" to "exhausted senescent" T cells: when markers predict functionality[J]. Cytometry A, 2014, 85(1): 25-35.

[17] Tomiyama H, Matsuda T, Takiguchi M. Differentiation of human CD8(+) T cells from a memory to memory/effector phenotype[J]. J Immunol, 2002, 168(11): 5538-50.

[18] Cibrian D, Sanchez-Madrid F. CD69: from activation marker to metabolic gatekeeper[J]. Eur J Immunol, 2017, 47(6): 946-953.

[19] Czystowska M, Gooding W, Szczepanski M J, et al. The immune signature of CD8(+)CCR7(+) T cells in the peripheral circulation associates with disease recurrence in patients with HNSCC[J]. Clin Cancer Res, 2013, 19(4): 889-99.

[20] Moschovakis G L, Bubke A, Friedrichsen M, et al. The chemokine receptor CCR7 is a promising target for rheumatoid arthritis therapy[J]. Cell Mol Immunol, 2019, 16(10): 791-799.

[21] Alarcon B, Berkhout B, Breitmeyer J, et al. Assembly of the human T cell receptor-CD3 complex takes place in the endoplasmic reticulum and involves intermediary complexes between the CD3-gamma.delta.epsilon core and single T cell receptor alpha or beta chains[J]. J Biol Chem, 1988, 263(6): 2953-61.

[22] Roman-Fernandez I V, Garcia-Chagollan M, Cerpa-Cruz S, et al. Assessment of CD40 and CD40L expression in rheumatoid arthritis patients, association with clinical features and DAS28[J]. Clin Exp Med, 2019, 19(4): 427-437.

[23] Bradley L M, Dalton D K, Croft M. A direct role for IFN-gamma in regulation of Th1 cell development[J]. J Immunol, 1996, 157(4): 1350-8.

[24] Gonzalez S, Groh V, Spies T. Immunobiology of human NKG2D and its ligands[J]. Curr Top Microbiol Immunol, 2006, 298: 121-38.

[25] Champsaur M, Lanier L L. Effect of NKG2D ligand expression on host immune responses[J]. Immunol Rev, 2010, 235(1): 267-85.

[26] Romagnoli P, Strahan D, Pelosi M, et al. A potential role for protein tyrosine kinase p56(lck) in rheumatoid arthritis synovial fluid T lymphocyte hyporesponsiveness[J]. Int Immunol, 2001, 13(3): 305-12.

[27] Yu X, Harden K, Gonzalez L C, et al. The surface protein TIGIT suppresses T cell activation by promoting the generation of mature immunoregulatory dendritic cells[J]. Nat Immunol, 2009, 10(1): 48-57.

[28] Zhao W, Dong Y, Wu C, et al. TIGIT overexpression diminishes the function of CD4 T cells and ameliorates the severity of rheumatoid arthritis in mouse models[J]. Exp Cell Res, 2016, 340(1): 132-8.

[29] Piedra-Quintero Z L, Wilson Z, Nava P, et al. CD38: An Immunomodulatory Molecule in Inflammation and Autoimmunity[J]. Front Immunol, 2020, 11: 597959.

[30] Wang H, Kadlecek T A, Au-Yeung B B, et al. ZAP-70: an essential kinase in T-cell signaling[J]. Cold Spring Harb Perspect Biol, 2010, 2(5): a002279.

[31] Gordiienko I, Shlapatska L, Kovalevska L, et al. SLAMF1/CD150 in hematologic malignancies: Silent marker or active player?[J]. Clin Immunol, 2019, 204: 14-22.

[32] Mcardel S L, Terhorst C, Sharpe A H. Roles of CD48 in regulating immunity and tolerance[J]. Clin Immunol, 2016, 164: 10-20.

[33] Li X, Yang Y, Sun G, et al. Promising targets and drugs in rheumatoid arthritis: a module-based and cumulatively scoring approach[J]. Bone Joint Res, 2020, 9(8): 501-514.

[34] Xiao L, Xiao W, Zhan F. Integrative Analyses of Biomarkers and Potential Therapeutic Drugs for Rheumatoid Arthritis[J]. Ann Clin Lab Sci, 2022, 52(1): 141-153.

[35] Xiong Y, Mi B B, Liu M F, et al. Bioinformatics Analysis and Identification of Genes and Molecular Pathways Involved in Synovial Inflammation in Rheumatoid Arthritis[J]. Med Sci Monit, 2019, 25: 2246-2256.

[36] Ge Y, Zhou L, Chen Z, et al. Identification of differentially expressed genes, signaling pathways and immune infiltration in rheumatoid arthritis by integrated bioinformatics analysis[J]. Hereditas, 2021, 158(1): 5.

[37] Ju S T, Panka D J, Cui H, et al. Fas(CD95)/FasL interactions required for programmed cell death after T-cell activation[J]. Nature, 1995, 373(6513): 444-8.

[38] Yildir S, Sezgin M, Barlas I O, et al. Relation of the Fas and FasL gene polymorphisms with susceptibility to and severity of rheumatoid arthritis[J]. Rheumatol Int, 2013, 33(10): 2637-45.

[39] Artukovic M, Ikic Matijasevic M, Markotic A, et al. FasL (rs763110) gene polymorphism is not associated with susceptibility to rheumatoid arthritis in Croatian population[J]. Croat Med J, 2020, 61(6): 547-555.

[40] Pundt N, Peters M A, Wunrau C, et al. Susceptibility of rheumatoid arthritis synovial fibroblasts to FasL- and TRAIL-induced apoptosis is cell cycle-dependent[J]. Arthritis Res Ther, 2009, 11(1): R16.

[41] Zhu A, Wang M, Zhou G, et al. Fas/FasL, Bcl2 and Caspase-8 gene polymorphisms in Chinese patients with rheumatoid arthritis[J]. Rheumatol Int, 2016, 36(6): 807-18.

[42] Mohammadzadeh A, Pourfathollah A A, Tahoori M T, et al. Evaluation of apoptosis-related gene Fas (CD95) and FasL (CD178) polymorphisms in Iranian rheumatoid arthritis patients[J]. Rheumatol Int, 2012, 32(9): 2833-6.
