# Supplementary material for: Identification of SLAMF1 as an immune-related key gene associated with rheumatoid arthritis and verified in mice collagen-induced arthritis model
Source: Front Immunol. 2022 Aug 30;13:961129. doi: 10.3389/fimmu.2022.961129 (PMC9468826; doi:10.3389/fimmu.2022.961129)
Supplement: Supplementary file 4 [file Table_2.docx]

**Table 2. Analysis and gate strategy for Flow cytometry detection**

| **Immune cells defined by ssGSEA** | **Immune Cells analyzed by flow cytometry** | **Gate strategy** |
| --- | --- | --- |
| Activated CD4 T cell | Th cell | CD3^+^CD4^+^CXCR5^-^CD25^-^TCRγδ^-^ |
| Type 1 T helper cell |  |  |
| Type 2 T helper cell |  |  |
| Central memory CD4 T cell |  |  |
| Effector memory CD4 T cell |  |  |
| Activated CD8 T cell | CTL | CD3^+^CD8^+^TCRγδ^-^ |
| Effector memory CD8 T cell |  |  |
| Central memory CD8 T cell |  |  |
| Regulatory T cell | Treg | CD3^+^CD4^+^CD25^+^ |
| T follicular helper cell | Tfh | CD3^+^CD4^+^ CXCR5^+^ |
| Gamma delta T cell | γδT cell | CD3^+^TCRγδ^+^ |
| Activated B cell | B cell | CD3^-^B220^+^CD19^+^ |
| Immature B cell |  |  |
| Natural killer cell | NK cell | NKp46^+^CD1d^-^ |
| CD56^bright^ natural killer cell |  |  |
| CD56^dim^ natural killer cell |  |  |
| Natural killer T cell | NKT cell | NKp46^+^CD1d^+^ |
| Macrophage | Macrophage/Monocyte | F4/80^+^ |
| Monocyte |  |  |
| Activated dendritic cell | DC | CD11c^+^ |
| Immature dendritic cell |  |  |
| Eosinophil | Eosinophil | Siglec^-^F^+^CD11b^+^ |
| MDSC | MDSC | Gr-1^+^CD11b^+^ |
